# Healthcare workers’ perspectives on trauma and mental health access for Rohingya refugees in Cox’s Bazar

**DOI:** 10.3389/fpubh.2024.1458680

**Published:** 2025-01-27

**Authors:** Lindsey Green, Linda Kaljee, Shahanoor Akter Chowdhury, Thomas McHale, Ranit Mishori, Dahlia Fateen, Nima Sheth

**Affiliations:** ^1^Physicians for Human Rights, Boston, MA, United States; ^2^Henry Ford Health, Global Health Initiative, Detroit, MI, United States; ^3^Georgetown University School of Medicine, Washington, DC, United States; ^4^Center for Mental Health Services, Substance Abuse and Mental Health Services Administration (SAMHSA), Rockville, MD, United States

**Keywords:** Rohingya, mental health, refugee mental health, humanitarian response, human rights, Myanmar, Bangladesh, sexual violence

## Abstract

**Background:**

In August 2017, the Rohingya population of northern Rakhine State in Myanmar fled to Bangladesh following “clearance operations” by the Myanmar security forces that were characterized by widespread and systematic violence, constituting severe human rights violations. The “clearance operations” were preceded by years of consistent denial of the human rights of the Rohingya people in Myanmar. This study examines the impact of these human rights violations on the trauma experiences of Rohingya survivors, their resulting mental health, and the availability and access to post-migration mental health services.

**Methods:**

Qualitative one-on-one interviews were conducted with 26 health care professionals who cared for Rohingya refugees after their arrival in Bangladesh.

**Results:**

Trauma experienced by the Rohingya spanned the period before, during, and following migration and was ongoing at the time of data collection. The impact of Rohingya survivors’ concurrent grief and trauma in relation to the violence they experienced in Myanmar as well as during and after their journey to Bangladesh that, at times, exacerbated survivors’ trauma presentation. There were limited mental health services available in Bangladesh and additional structural and procedural barriers to care that limited responsiveness to Rohingya survivors’ mental health needs.

**Conclusion:**

The Rohingya experienced traumatic human rights violations in Myanmar that led to their forced migration to Bangladesh. The Rohingya continued to experience trauma during their forced migration to Bangladesh and were exposed to additional stressors in the post-migration settings in refugee camps, including lack of access to adequate mental health services. These experiences have resulted in a spectrum of stress-and trauma-related symptoms. These findings show the mental health impact of protracted human rights violations on the Rohingya, revealing how trauma is not a singular event but a continuous experience. Detailing the limited mental health infrastructure and structural barriers facing Rohingya refugees in Bangladesh, the findings underscore the urgent need for trauma-informed interventions that address the complex psychological consequences of systemic violence and displacement. The findings emphasize the critical importance of holistic mental health support in refugee settings, providing evidence-based recommendations for the public health and humanitarian sector to consider when designing programs to address the mental health and psychosocial support needs of sexual violence survivors living in conditions of displacement.

## Research context

Current frameworks for understanding mental health outcomes for displaced and conflict-affected populations consider pre-migration, migration, and post-migration stressors as contributors to mental health outcomes and overall well-being (1, 2). Olcese et al.’s scoping review on migration and community resilience underscores that migrants face profound economic, social, and psychological hardships, which are further intensified by experiences of violence or displacement before migration (3). Resilience-building within these communities depends heavily on social capital, community competence, and adaptive attitudes, emphasizing the critical role of host contexts in fostering recovery. For refugee populations like the Rohingya, these insights underscore the need to address structural and systemic barriers while strengthening communal support mechanisms to mitigate cumulative stress and enhance mental health outcomes.

In August 2017, Myanmar security forces, accompanied by local civilians, launched “clearance operations” against the Rohingya population of northern Rakhine state (4). Serious human rights violations were widespread and systematic, including shootings, beatings, sexual violence, and destruction of property (4–8). Such human rights violations had been preceded by years of persistent human rights violations, including denial of citizenship, restrictions on movement, forced displacement, denial of the right to health and education, lack of religious freedom, looting and destruction of property, arbitrary and illegal detentions, killings, forced labor, and trafficking (5, 9, 10).

Following the ‘clearance operations,’ more than 720,000 Rohingya refugees^1^ fled to Bangladesh. After a long, treacherous journey, Rohingya refugees settled in camps near Cox’s Bazar. These camps quickly grew to become the largest refugee settlement in the world which put enormous strain on the existing social, cultural, and economic environment and infrastructure in this area of Bangladesh (11, 12). For the Rohingya, prolonged exposure to gross violations of human rights in Myanmar and on-going, systemic discrimination, deprivation of basic rights, and considerable safety concerns in Bangladesh, have increased their vulnerability to poor mental health outcomes (2, 13).

Challenges related to basic living conditions in within the refugee camp setting include access to health care and continued exposure to violence and crime and conflict. These challenges were exacerbated by the COVID-19 pandemic (14–20). In addition, the ongoing humanitarian crisis worsened barriers preventing access to essential health care services for women, girls, children and other vulnerable populations leading to increased distress and negative impacts (18, 21).

The Rohingya population in Bangladesh has experienced significant stressors related to the on-going and evolving political situation in Myanmar and limited prospects for return. In February 2021, the Myanmar miltary *coup d’état* and the ensuing violence and repression, have intensified the suffering of Rohingya and other minority communities within the country and caused significant social, political and economic instability (22–24). Increasing conflict since 2022 between the Myanmar Armed Forces and Arakan Army in Rakhine State has led to transportation blockages, displacement, and reduced humanitarian access, affecting livelihoods and increasing malnutrition rates (25). In the central region of Rakhine State, numerous internally displaced persons (IDPs) are compelled to return to areas that still pose significant protection and security risks (26). Due to ongoing insecurity, limited access to basic services, and the closure of IDP sites, some individuals are likely to experience multiple displacements (27–31).

Studies emphasize that the links among pre-migration trauma, displacement stressors, and post-migration living conditions to mental health outcomes, including PTSD and depression, are well-established (2, 32, 33). The Rohingya population have experienced prolonged exposure to violence, human rights violations, and statelessness, which are further compounded by post-migration adversities (15, 34). The accessibility and adequacy of mental health services in the post-migration context, particularly within refugee camps, remains underexplored. There is also limited research on healthcare workers’ perspectives to support more nuanced investigations into service gaps and tailored interventions.

With an understanding of Rohingya refugees’ experiences of forced migration and the particular mental health challenges faced by displaced, this study sought to capture trauma experiences in the periods before, during and after Rohingya survivors’ migration to Bangladesh and their resulting mental health presentations. The authors collected these data by speaking to health care workers who provided services to Rohingya survivors in Bangladesh to understand both the trauma experiences of survivors and post-migration mental health services within the Rohingya refugee camps during the period of data collection and analysis.

## Materials and methods

Qualitative one-on-one semi-structured interviews were conducted with health care professionals who cared for Rohingya refugees in Bangladesh between 2017 and 2020. The focus of the overall research project was to understand health care professionals’ experiences providing care to Rohingya survivors with an emphasis on patterns of perpetration of sexual violence and access to health care. In addition, data were obtained related to the impact of the COVID-19 pandemic on services for Rohingya survivors (8, 20). The data presented and analyzed here are specific to mental health presentation and access to services and represent a sub-set of the findings from this larger research project.

The study was intentionally designed to gather information from healthcare workers about the experiences of Rohingya refugees without directly interviewing survivors to avoid subjecting them to potential re-traumatization. Health care workers, through their clinical evaluations and interactions with numerous patients, can provide unique insights into both individual cases and can provide data to support overarching patterns and trends in survivors’ experiences. In addition, health care workers can provide information about both short-and long-term after-effects of trauma, offering a more comprehensive understanding that might not be available through direct survivor interviews alone.

The research team included social scientists, public health experts, and physicians from the United States and Bangladesh. Data were collected between November 2019 and August 2020. Due to several barriers, including the Covid-19 pandemic, all interviews but one were conducted virtually using Zoom, Skype, and phone. The in person interview was conducted in Cox’s Bazar with a participant still working there. Interviews were conducted in English and Bengali. All interviewers were established qualitative researchers and public health experts with extensive experience working on projects focused on sexual and reproductive health, conflict-related sexual violence, orphaned and vulnerable children, and displaced persons.

### Study population

A purposive sampling strategy was used to recruit health professionals who fit the inclusion criteria: a healthcare worker of any nationality and any discipline and specialty who worked with Rohingya refugees in camps in Cox’s Bazar in Bangladesh between August 2017 and August 2020. Identification of participants included outreach to known contacts working with the Rohingya during the inclusion period and recommendations from those contacts and other professionals working in Cox Bazar. The cohort included physicians, nurses, midwives, community health workers, mental health and psychosocial support workers, case managers, SGBV and protection workers, and health volunteers. As is standard practice with qualitative research, data saturation, which is the point at which new themes or information cease to emerge from the data, was used to establish the final sample size (35). To help determine the point of ‘data saturation’ regular conference calls were conducted to provide an opportunity for the study team to discuss preliminary findings, data gaps, and identify potential participants to help fill in those gaps.

No compensation was provided for participation in the interview. All participants provided informed consent prior to starting interviews.

### Data instruments

Data was collected via a semi-structured interview guide and a demographic form. The interview guide covered key areas, including professional background and contextual details of work with the Rohingya, general experiences treating Rohingya patients, experiences specific to physical violence, SGBV, and mental health status, and challenges in addressing trauma and health care. A summary of main themes for the interview were provided to respondents in advance of the interview.

All interviews were audio recorded and transcribed. Interviews in English were conducted by US-based researchers and transcribed verbatim by a professional service. Interviews in Bangla were conducted by a Bangladeshi researcher, fluent in both Bangla and English. These interviews were transcribed in Bangla and then translated into English with quality checks for accuracy. Transcribed data were uploaded into a qualitative data management program (Dedoose) (36). All transcripts were analyzed in English. The research team created a coding dictionary to ensure data were coded consistently by four study team members from the United States and Bangladesh. An intercoder reliability assessment was conducted on a sample of transcripts which indicated an overall consistency in the use of the codes. In addition, research team members met regularly during the data analysis project to review coding and determine if new codes were required for emergent themes. After completion of data coding, themes and sub-themes were identified and reviewed by the team. Illustrative examples of the themes and sub-themes were selected from the text. All interviewers were established qualitative researchers with extensive experience working on projects focused on sexual and reproductive health, orphaned and vulnerable children, and displaced persons.

The study received ethical approval from the Georgetown University IRB (STUDY00001282 and MOD00004144) and an exemption was given by PHR’s Ethical Review Board. Permission to conduct the study was also received from the Office of the Refugee Relief and Repatriation Commissioner (RRRC) in Cox’s Bazar, Bangladesh.

### Limitations

This research has several limitations. The study relied on health professionals’ experiences and observations of the physical and mental health status of a population that is different from them culturally and linguistically. While the study population represented different geographic, professional, and cultural backgrounds, it did not include Rohingya health care providers.

Observations must therefore be viewed as highly contextual and potentially affected by biases. The survivors’ stories recounted in these interviews were communicated through multiple cultural and linguistic filters, including interpretation. Additionally, our cohort was selected via snowball sampling and is not representative of all health professionals who worked in the camps.

Interviews were conducted in 2020, in some cases more than 3 years after respondents had had the experiences that they had described to the interviewers. Recall bias, therefore, must be considered. However, respondents were given a summary of the main research themes prior to the interview and some respondents reviewed written notes or journals from their time spent in Cox’s Bazar during the interviews. Clinicians who had worked in the camps in August or September 2017 may have observed the manifestation of more acute trauma, suggesting potential chronological bias. Participants interviewed had a wide range of time periods when they supported Rohingya survivors, some had been part of an acute response during 2017 and 2018 while others were still working with Rohingya refugees in Bangladesh at the time of the interview. This variety in participant experiences allowed us to observe more about the acute and long-term mental health challenges for Rohingya survivors.

Lastly, while some of the respondents interviewed were health care professionals with mental health specialization or experience, most respondents were not specialists in psychiatry, behavioral, or mental health.

Despite these limitations, there were significant similarities across the collected narratives reflecting on the experiences of the Rohingya.

As a qualitative study, the interpretation and analysis of data is subject to interpretation biases introduced by the researchers. The research team was multidisciplinary, drawn from a variety of cultural backgrounds, and worked collaboratively to address potential biases in the interpretation of results.

## Results

### Demographics

We recruited 26 health professionals who fit the inclusion criteria. Table 1 describes their characteristics.

**Table 1:** Demographic information for health worker respondents.

Respondents	*n =* 26
Respondent gender	
Female	20
Male	6
Respondent age	
25–34 years old	11
35–44 years old	10
45–54 years old	2
Over 55 years old	3
Respondent nationality	
Bangladesh	11
United States	9
Australia	1
Japan	1
Malaysia	1
United Kingdom	1
United States/Australia	1
United States/Canada	1
Respondent place of employment during period working with Rohingya refugees	
NGO	25
Multilateral organization (e.g., UN, WHO)	1
Respondent position while working with Rohingya refugees	
Physician	12
Nurse	2
Nurse midwife	1
Nurse practitioner	2
Clinical psychologist	1
Case manager	3
Paramedic	1
Other	4
Period in which respondent worked with Rohingya refugees in Bangladesh*	
2017	14
2018	21
2019	10
2020	4

*Number of respondents known to be working with Rohingya refugees in that calendar year.

### Trauma experiences

Respondents recognized that Rohingya refugees’ trauma experiences were ongoing and spanned pre-, during, and post-migration periods. On arriving in the camps, however, health professionals tried to address Rohingya patients’ immediate health needs while recognizing the impact of their past and concurrent experiences of trauma and grief. Table 2 provides examples of the different phases during which Rohingya survivors experienced trauma, as noted by the respondents.

**Table 2:** Phases and types of traumatic experiences of Rohingya refugees who settled in camps in Bangladesh.

Migration phase	Examples of traumatic experiences
Pre-migration	Destruction of property Violence Sexual violence Witnessing violence Forced displacement
Migration	Injuries sustained during journey Drownings Physical violence Disease Food insecurities
Post-migration	Sexual violence Intimate partner violence Instability in Bangladesh Lack of sanitation and basic needs in the camp

### Pre-migration experiences

Health care workers were well-aware of the multiple traumas experienced by Rohingya refugees prior to leaving Myanmar. The health care workers described being alert to potential for their patients to disclose violent experiences, including those related to the destruction of villages, and killings.

We all knew they were fleeing Myanmar because there were the atrocities that were happening there. They were being killed, their villages were being burned, their homes were being burned..” A nurse working in Cox's Bazar in 2017.

“It was kind of like the understanding that everybody coming in had witnessed,… if not experienced, some level of abuse…. So, we were always on heightened alert looking for those types of things.” A nurse working at a primary health clinic in Kutupalong camp in 2018.

Health care workers also described specific instances in which patients or family members of patients described experiences of witnessing physical violence and death. Because of stigma, many health care workers’ heard indirect accounts of sexual violence from their patients or saw signs of sexual violence from those who did not disclose a history of sexual violence, such as from women coming into the clinic for to treat sexually transmitted infections or unwanted pregnancies.

One case … was this 11-year-old who came in because her mom said she stopped talking. That was striking to me because she had basically selective mutism as a manifestation of her PTSD. I suspected she was probably either raped or had some sort of gender-based violence, but she denied it. Then I interviewed the mom separately and found out that, actually, her father, his legs were chopped off in front of her by the military. A fellow Rohingya man saw and took the father, and they fled across the border into Bangladesh, and they took him immediately to the field hospital, and they have not seen him since, and that was like months ago …. She stopped talking since that experience.” A volunteer physician working in Cox’s Bazar in 2018.

“I did have an 11-year-old girl come in who brought in her … five-and seven-year-old [siblings].. She was the ‘mom.’ Both parents had been killed.” A pediatrician working in Cox’s Bazar in 2017 and 2018.

### Migration journey experiences

The arduous journey from the Rohingya refugees’ home villages in Myanmar to Bangladesh required several days of walking through difficult terrain and across the river border between the two countries. Refugees left their homes with no provisions and suffered injuries, illnesses, and death during migration. Families lost contact with one another including parents and their children (37). Health care workers heard about these experiences from their patients.

There were a lot of people who had like actual physical injuries from the journey or from the trauma and the events that were preceding that. We would see someone that had a laceration that they said was from a machete from that conflict or someone that was injured in the process of migrating, like while they were walking for forever without shoes.” An emergency nurse working in satellite health clinics in December 2017.

We saw lots and lots of parents or moms who had lost many kids who would say, ‘I had five kids trying to cross the river. This is the only one I could hold.’” A pediatrician working in Cox's Bazar in 2017 and 2018.

“I would say everybody.. we saw [was] suffering from the effects of the violence and the trip altogether.” A nurse working in Cox's Bazar in 2017.

### Post-migration experiences

The influx of almost 800,000 persons into Bangladesh over a short period of time meant that local, national, and international actors where not initially prepared for the arrival of this many people. The crowded Cox Bazar refugee sites were without basic sanitation and survival needs, including water and food. Access to healthcare was limited with a focus on immediate needs for those injured or suffering from infectious disease outbreaks including measles and diphtheria. In addition, this large population had endured extreme physical, psychological, and physical trauma, requiring complex, long-term care and treatment (38). On arrival and even years into resettlement, the Rohingya continued to experience sexual violence, intimate partner violence, and multiple physical and mental health challenges exacerbated by poor access to healthcare and economic, social, and political instability (39).

“I definitely saw gender-based violence. It was often within the community, from the camp itself, within the community…” A physician working with Rohingya refugees in 2017 and 2018.

“Hitting, punching, kicking on any part of the body, including the head, and even if you're pregnant, it could still be the abdomen. …. We've had women whose husbands have tried to hang them, whose husbands have tried to poison them, who have been burned. Yeah, we see all kinds but hitting and kicking and punching are definitely the most common.” A nurse practitioner working at an outpatient clinic in Kutupalong in 2017.

“I didn't see any domestic violence in November, but as the time goes on.. end of December or beginning of January we started to see some domestic violence.. The stress shows.. The direction of stress comes into the, inside of the house because the life there was very difficult.” A nurse midwife working in Kutupalong camp in 2017.

“They've moved from kind of crisis mode, from the things that happened there. It certainly still affects them, and they still think about it, but that's not why they're coming to the clinic.. They still bring it up sometimes during their visits, but it's more of the trauma that we would see now would be related to current situations.” A nurse working in Cox's Bazar in 2017.

### Presentation and consequences of trauma

While the violence sometimes left physical injuries and scars, other forms of trauma, such as sexual violence and psychological trauma, were primarily evident through the stories told to the health care providers during care for other conditions. In addition, health care workers experienced (Table 3).

**Table 3:** Presentation and consequences of trauma experienced by Rohingya.

	Examples of presentation and consequences
Presentation of Trauma	Lack of affect Lack of appetite Somatic complaints Mutism Associated physical conditions, e.g., STIs
Consequences of Trauma	Depression, anxiety Panic attacks Hypervigilence

When survivors shared their experiences with health professionals, the associated psychological trauma was sometimes expressed to them by patients explicitly, and at other times it was discernable from their behaviors, including their affect or lack of engagement or responsiveness.

“The thing that you did see was just overall deep, deep sadness in these women. The women would come in … [covered] …, so you could only see their eyes, but many of them, when I'd be taking care of their kids, would start to cry.” A pediatrician working in Cox's Bazar in 2017 and 2018.

Providers reported seeing a wide range of mental health and trauma related symptoms including hypervigilance, emotional numbing, re-experiencing, avoidance, lack of appetite, and co-occurring symptoms of depression, anxiety, and panic attacks.

“We had patients present regularly enough, like complaining of depression, anxiety, panic attacks, those were all fairly common complaints.” An emergency nurse working in satellite health clinics in December 2017.

Clinicians shared that patients regularly presented with somatic complaints, including physical pain, that the clinicians attributed to their psychological distress.

“A huge number of our patients had somatic complaints, like very vague. Like, ‘I've had a headache or a stomachache or backache.’ And then when you dig deeper into how long [they] have these things … it's been going on as long as they left their home or as long as they've been living in the camps. … we couldn't find anything wrong with them clinically and it seemed like the providers would attribute it to just a stress reaction that manifested in a physical way. And that was very, very common.” An emergency nurse working in satellite health clinics in December 2017.

Some survivors experienced such severe trauma responses that they stopped eating and speaking.

“And then there was a 15-year-old who had been raped and was mute and mostly catatonic. She wasn't talking, she wasn't eating. She had also seen her brothers killed and she had been raped. … Our clinic did have a psychologist, so when a patient came with severe problems and they didn't seem to have any physical problems, then we would have them come back every day and talk to a counselor. But this child was much too sick to stay with us.” A nurse working in Cox's Bazar in 2017.

Patients often disclosed trauma and traumatic experiences associated with sexual violence when seeking care for a wide range of services, including sexual and reproductive health.

“And then there was a woman who … had complaints of vaginal discharge. So, I asked her if I could examine her vaginally. And then when I did the exam it looked like she had some trauma, just from the scars on her perineum. So, I asked her a few questions about that, and then she started crying and talked to me about her experience of rape at the hands of the military, the Myanmar military… she and I think around 14 other women had been taken and locked into a house, and that they were all gang raped. And some of them did not survive, but she was able to survive.” A nurse practitioner working at an outpatient clinic in Kutupalong camp in 2017.

Clinicians also reported that patients who were seeking sexual and reproductive health care disclosed trauma from witnessing sexual violence, often against a relative.

“I remember an elderly lady who came in just with a bladder infection, a UTI. And then somehow the conversation devolved into the fact that she had watched her daughter-in-law get gang-raped by six soldiers in their home.” A volunteer physician working with Rohingya refugees in January 2018.

### Services for mental health care and barriers to care

At the start of the humanitarian response in Cox’s Bazar in late 2017, there was limited mental health capacity within the health sector, especially a lack of inpatient capacity for acute or severe mental health case management. Respondents described numerous barriers that limited access to care and responsiveness to the refugees’ mental health needs. These barriers likely contributed to poor post-migration mental health outcomes. Barriers identified in this study included structural, legal, and medical/procedural issues.

Table 4 provides examples of the types of barriers faced by Rohingya refugees when seeking mental health services and support.

**Table 4:** Barriers to mental health care among Rohingya refugees settled in camps in Bangladesh.

Type of barrier	Examples of barriers
Structural and Legal	Restricted movement outside of camps Bureaucratic hurdles to accessing care outside of camps Size and remoteness of camps
Medical and Procedural	Physical structures of clinics Daily workload for health care providers/time spent with each patient Limited number of health care workers with specific mental health training Limited/no pathways for referral or follow up Screening protocols not validated for Rohingya culture and language

### Structural and legal barriers

Respondents commented on the restrictions Rohingya refugees faced in their ability to leave the camps due to a lack of legal status in Bangladesh. These restriction affected access to health services and contributed to a sense of confinement and isolation. The size and number of refugee camps also limited access to care, sometimes requiring survivors to prioritize traveling to remote service points over other demands on their time such as accessing food or clean water.

“The nearest clinic that would offer mental health services by trained professionals would be a 20-minute walk from our clinic, so sometimes it's just too much effort.” A nurse practitioner working at an outpatient clinic in Kutupalong camp in 2017.

### Medical and procedural barriers

Many respondents noted the lack of private space for discussions about sensitive issues and/or for counseling sessions.

“It is very tough when we try to make confidentiality and rapport building, and sometimes [if] there is any secret issue. Lots of noises, so clients sometimes feel discomfort to give enough information …. Besides the room, there is another room, they are talking, working and even they are laughing sometimes …” A clinical psychologist working in Cox's Bazar in 2018.

For health care workers, long workdays in the clinics and high daily patient loads significantly impacted their time with each patient and thereby limited the time available to discuss mental health and SGBV issues or to conduct screenings and referrals.

“I think that some of the challenges are time. If I had time, I probably would have asked the trauma stories from every one of the patients because I think that's part of healing. I think that time was definitely a great limiting factor.” A volunteer physician working in Cox's Bazar in 2018.

Some healthcare workers also identified continuity of services for survivors as a barrier. Agencies providing services experienced inconsistent funding streams and, health care providers often served on short-term emergency response assignments. Available health services also had little to no access to providers with mental health training. Healthcare workers felt that inadequate resources and infrastructure, including telecommunication outages and blockages, limited their ability to make appropriate referrals and follow up with patient’s progress.

“The funding was always like short-term, very short-term funding. Like one-month, two-month, maximum three-month funding. So that hampered our program planning and design.” A public health administrator for a multilateral organization in Cox's Bazar in 2017.

Many respondents shared that standardized mental health screening protocols were not adapted for the Rohingya culture nor translated into the Rohingya language. These tools were also perceived to be too long and complex for use within the refugee health settings. One respondent identified challenges with the integration of mental health screening protocols during a clinical encounter, especially when patients presented with multiple health issues that needed to be addressed in and environment where follow-up visits were not guaranteed.

“I question where the most appropriate place to deploy the screening tool would be as far as like at what point during their exam? Typically, a lot of screening tools are kind of used towards the beginning …. If they have decided that they're going to come in with kind of generalized abdominal pain as a complaint and you ask them about sexual violence kind of before you'd gotten to that they might feel uncomfortable jumping the gun.” An emergency nurse working in satellite health clinics in December 2017.

Within the complex and limiting service delivery context, some health care professionals perceived that giving their patients a safe space to discuss their experiences was important, even while recognizing the limits of their ability to provide care.

“That's why my personal philosophy was if I had a minute or two or if I had even a little bit of extra time to spare, I would want to kind of serve as sort of a counselor role, even though that's not my training …. I'm not a psychologist, I'm not a clinical psychiatrist, but I felt like I had to act like one for a lot of the patients because they're coming in with vague symptoms like … muscle aches or stomach pain. That's sometimes somatization of trauma.” A volunteer physician working in Cox's Bazar in 2018.

Respondents also described the challenges faced in obtaining information from female survivors about sexual health, SGBV, and associated mental health when a majority of interpreters and providers were male. Additionally, they noted that it was often difficult for interpreters to discuss and translate questions and language related to mental health conditions to patients because of a lack of cultural understanding of mental health and medically trained interpreters.

“Probably it was just there and not disclosed for [a] multitude of reasons. Number one, I'm a western man, western white man speaking with a Rohingya woman patient and even if there was just kind of a simple gynecological complaint, many Rohingya women would prefer just to continue with their complaint than allowing a strange man to examine them, even with another woman in the room …. we wanted to train female English interpreters … [but] those female interpreters and their families were threatened with physical violence if they allowed their daughters to go be trained to be an English translator in the clinic.” An outpatient attending physician in Kutupalong camp in 2018 and 2019.

## Discussion

Health workers play a critical role in addressing the mental health challenges of forcibly displaced populations by serving as both frontline responders and community liaisons. Their responsibilities often extend beyond conventional care to include trauma-informed practices, cultural competence, and advocacy for improved access to essential health services. Given the intersection of migration-related stressors with pre-existing trauma, health professionals are uniquely positioned to mitigate the compounded psychological distress experienced by refugees. For example, they deliver culturally sensitive psychosocial interventions, often adapting to resource-scarce environments to provide meaningful support. In addition, health workers can help bridge the gap between displaced populations and host communities, promoting integration and resilience.

Interview data from health workers confirms previously documented information about the traumatic human rights violations in Myanmar that led to the forced migration of the Rohingya. These interviews further provide important data related to traumas associated with the migration journey and experiences post-migration within the Cox Bazar camps.

This study offers an important contribution to the recent literature that seeks to document the continuum of trauma experiences and associated mental health outcomes for refugee populations as well as barriers to general health care and mental health care in post-migration settings (13, 21, 34, 40–42). The study also provides a structured, phased approach to assessing these experiences and identifying the factors that contribute to Rohingya refugees’ psychological, emotional, physical, and social well-being. These data also provide insights into the logistical, economic, social, and cultural challenges in providing health care in the context of a large influx of persons in urgent need of a broad spectrum of medical interventions (43–47). In addition, through the perspectives of health care professionals, mental health services can be developed and adapted to address some of these limitations within refugee settings that negatively affect delivery and accessibility and acceptability.

### Assessing Rohingya experiences

Within the context of clinical care, many Rohingya refugees disclosed traumatic experiences, including sexual violence and killing of family members, to health care workers. In other instances, these experiences were disclosed by family or community members. In some cases, trauma was presented either somatically with physical pain or through subtle interpersonal changes such as lack of engagement or responsiveness. It is important to note that these specific manifestations of trauma may only capture a small fraction of trauma experiences within this population. However, the reported presentations of trauma are consistent with what would be expected and have been documented elsewhere (48).

Healthcare workers also helped to provide data on the multiple physical and psychological trauma associated with the migration process. These data are important to understanding the continuum of events which impact the mental health of the Rohingya and other refugees including the migration experience, on-going social and political upheaval within the Rohingya’s home country, and post-migration experiences within Bangladesh (49).

The instability in the post-migration environment of the Rohingya, from their stateless immigration status to scarcity of resources to dismantled social networks, left the community with added stress, continuous fear and a shared, collective, experienced trauma that affects the social fabric of the community (13, 50). Not having proper channels to address these experiences could have contributed to various other stressors, such as intimate partner violence, increased burden on children to assume care responsibilities, and suppression of pre-migration traumas for fear of social stigma. The continued trauma experienced by many likely affected their ability to readjust and find stability as individuals and as a group (13, 15, 21, 40, 51).

As noted, the narratives shared by the health care workers are consistent with other studies that have found mental health conditions to be prevalent among the Rohingya refugee population and with frameworks that outline factors that contribute to refugee mental health (5, 13, 21). In one study, Rohingya refugees in Bangladesh compared to those in Malaysia were more likely to experience PTSD, depression, anxiety, and functional impairment. Data from the Cox’s Bazar Panel Survey indicated 30.0% of respondents reported depressive symptoms and 4.9% reported symptoms associated with PTSD (15, 51). The healing of Rohingya refugees should be viewed within a context of potentially compounded and continued trauma at both the individual and community level (52). A study analyzing household survey responses of Rohingya refugees in Bangladesh from 2019 and 2020 revealed a high prevalence of severe post-traumatic stress symptoms (PTSSs), particularly among those who experienced physical or sexual abuse before displacement, with 46.6% reporting severe PTSSs (42). Findings from this study however also indicated that adequate humanitarian aid and opportunities for paid employment in refugee camps mitigated risks of PTSSs related to post-migration stressors.

Outcomes from this study can be used to further research and humanitarian efforts among other populations. The experiences of the Rohingya, although unique in many ways, are not entirely dissimilar from the experiences of other forcibly displaced populations including Bosnian, Cambodian, Cuban, Iraqi, Sudanese, Syrian, and Tamil communities. In each of these communities, the continuum of migration stressors may either compound or be mitigated by interventions (52–55).

### Mental health services

This study substantiates reports about the lack of mental health infrastructure in Rohingya refugee camps in Bangladesh, especially during the initial humanitarian response, and the enormous difficulty accessing appropriate services. While improvements have been made to infrastructure, and more mental health services and programs have been added over time, there continues to be an inadequate mental health response (41). In a recent study, ongoing challenges to the availability of mental health services for Rohingya include inadequate numbers of skilled service providers and services for severe mental illness, lack of programs focused on children, and issues related to privacy and confidentiality of clients (56). In addition, Rohingya refugees ability to access to health services continues to be hampered by linguistic and socio-cultural barriers including perceptions and definition of health and illness, and lack of trust and fear of authority (57).

Evidence-based intervention approaches in the post-migration period may offer more effective and prompt healing spaces and should be explored to mitigate on-going stressors within the Rohingya community. Frameworks exist to address mental health issues in varying contexts (58). Leveraging multicomponent interventions that address survivors’ symptoms, culture, manifestations of trauma, social settlements, adaptations and interpersonal connectivity best support recovery for displaced populations with complex trauma exposure (59–64). Mental health and psychosocial support (MHPSS) services in the Rohingya humanitarian response have provided critical support to the Rohingya refugees However, the success of these interventions has been contingent on adaptation to account for the type of trauma experienced by the survivors, their symptoms, delivery logistics, and the socio-cultural and political contexts from which they came and to which they migrated (58, 59).

Interventions that should be prioritized for Rohingya survivors should be those that have worked best for other displaced populations in limited resource settings (59–64). In general, these interventions are characterized by: (1) low intensity mental health interventions that can be delivered to a variety of people with a wide range of diagnoses (trans-diagnostic), and (2) implementation through trained professionals with different skill levels (task-shifting) (61). Some of these interventions have been adapted in Rohingya populations including the Adaptation and Development After Persecution and Trauma (ADAPT) model, a version of which has been adapted for Rohingya refugees in both Malaysia and Bangladesh (60). Other interventions that should be considered are outlined in Table 5.

**Table 5:** Psychosocial interventions for trauma-affected groups in low-resource settings.

Intervention	Description
Adaptation and Development After Persecution and Trauma (ADAPT) model	The ADAPT model is targeted toward populations that have experienced mass conflict and offers a therapeutic intervention leading to improved adaptive capacity and resilience compared to the traditional and individualized Cognitive Behavior Therapy (CBT) (66).
Self Help Plus (SH+)	The lowest intensity intervention which focuses on stress management and coping skills (62). It is derived from Acceptance and Commitment Therapy (ACT), which is an effective therapy stemming from CBT. SH+ is far reaching and does not require mental health experts to be implemented and can be delivered to groups as large as 20–30 people. Importantly this intervention is applicable to survivors of sexual violence, is psychologically safe for those with and without mental health conditions (62).
Problem management Plus (PM+) Early Adolescent Skills for Emotions (EASE)	Based on CBT, PM+ and EASE, the children’s version, are similar transdiagnostic interventions meant to help individuals in low resources settings facing adversity (61). PM+ largely focuses on problem solving and behavioral activation strategies that can be more easily learned and delivered by lay individuals (61).
Group Interpersonal Therapy (IPT)	The WHO now gives guidance on a manual consisting of an eight-session protocol that guides supervised non-specialized staff in implementing group IPT) (67). Group IPT was found to be effective for depression in internally displaced adolescents in war-torn areas of Uganda (63).
Thinking Healthy	The WHO has also put out the Thinking Healthy manual for perinatal depression, which consists of recommendations on how CBT can be delivered effectively by community health workers (68).
Common Elements Treatment Approach (CETA).	CETA is an intervention that can be used in low resource settings and can be provided by trained and supervised lay providers. It combines a range of treatment options into a simplified structured guide that is ultimately customized by the provider (69).

These interventions are designed to be easy to implement and scale up in low-and middle-income countries. However, medication management and individual trauma approaches are necessary to address the needs of those with a higher degree of psychopathology (59). It is also important to assess implementation conditions including stakeholder engagement, cultural adaptation, current barriers to care, and staff training, supervision, and monitoring. Special challenges can arise with high staff turnover and lack of sustainable supervision systems, all of which exist in the context of mental health support for Rohingya survivors (60).

Training health workers in trauma-informed care, survivor-centered approaches, cultural competence, and trust-building with refugee communities is essential to mitigate barriers such as stigma, linguistic challenges, and lack of trust. Programs like Self Help Plus (SH+) and Problem management Plus (PM+), which have proven effective in low-resource settings, should be tailored to address Rohingya-specific experiences, such as their collective trauma and intergenerational suffering (61, 62). Furthermore, sustainable support systems for health worker supervision and capacity-building are crucial to maintain service quality and address challenges like staff burnout and turnover. Stakeholder engagement, cultural adaptation, and strengthened inter-agency coordination should also be prioritized to create a holistic, accessible, and culturally resonant mental health response.

## Conclusion

Focusing on pre-migration traumatic events in displaced populations can undermine the significance of traumas experienced during migration, the conditions in which displaced populations live, and how these conditions and post-migration stress, may also negatively impact mental health (65). Analyzing the mental health outcomes of displaced populations therefore necessitates a multi-pronged approach, acknowledging the nuanced experiences of such populations pre-, during, and post-migration.

This study shows persistent trauma related to experiences of conflict-related sexual violence years after the events of 2017 and offer insight into the long-term manifestations of trauma related to human rights violations experienced by Rohingya populations. Seven years since Rohingya survivors first experienced displacement and forced migration, this study demonstrates that the drivers for persistent trauma still exist, as do the opportunities to leverage existing models of mental health and psychosocial support for healing.

The findings of this research are consistent with well-documented and expected outcomes for forcibly displaced populations who have experienced trauma, violence, and prolonged displacement. These findings emphasize the importance of providing immediate low-resource psychological interventions as well as long-term individual trauma-focused psychotherapy for forced migrants to address pre-, during, and post-migration trauma.

This study highlights the ongoing mental health challenges faced by Rohingya refugees, stemming from traumatic pre-migration events, continued violence during migration, and persistent stressors in post-migration environments. To address these issues, healthcare workers and policymakers must prioritize culturally sensitive mental health services, such as Group Integrative Adapt Therapy (IAT-G) and Problem Management Plus (PM+), tailored to the unique needs of this population. Expanding access through mobile clinics, increasing the availability of trained professionals, and adopting task-shifting approaches can improve service delivery. Additionally, embedding mental health care into broader humanitarian assistance frameworks, supported by sustainable funding and inter-agency coordination, is essential to reduce barriers and foster resilience. These interventions can significantly enhance the well-being of Rohingya refugees and other displaced populations.

Findings from this study should be used to advocate for effective mental health services for Rohingya refugees specifically, and displaced populations more generally.

## Data Availability

The datasets presented in this article are not readily available because of the studies informed consent process. The deidentified data used for this analysis can be made available upon reasonable request, in accordance with the study’s informed consent process. Requests to access the datasets should be directed to lgreen@phr.org.

## Footnotes

^1^The Government of Bangladesh refers to the Rohingya as "Forcibly Displaced Myanmar Nationals" (FDMN).
